# T1 glottic laryngeal cancer: the role of routine follow-up visits in detecting local recurrence

**DOI:** 10.1007/s00405-021-06983-3

**Published:** 2021-08-06

**Authors:** Pihla Pakkanen, Taru Ilmarinen, Elina Halme, Heikki Irjala, Petri Koivunen, Matti Pukkila, Sami Ventelä, Jaana Hagström, Leena-Maija Aaltonen

**Affiliations:** 1 Department of Otorhinolaryngology-Head and Neck Surgery, University of Helsinki and Helsinki University Hospital, P.O. Box 263, 00029 Helsinki, Finland; 2 Department of Otorhinolaryngology-Head and Neck Surgery, University of Tampere and Tampere University Hospital, Tampere, Finland; 3 Department of Otorhinolaryngology-Head and Neck Surgery, University of Turku and Turku University Hospital, Turku, Finland; 4 Department of Otorhinolaryngology-Head and Neck Surgery, University of Oulu and Oulu University Hospital, Oulu, Finland; 5 Department of Otorhinolaryngology-Head and Neck Surgery, University of Eastern Finland and Kuopio University Hospital, Kuopio, Finland; 6 Department of Pathology, University of Helsinki and Helsinki University Hospital, Helsinki, Finland; 7 Department of Oral Pathology and Radiology, University of Turku, Turku, Finland

**Keywords:** Glottic cancer, Early laryngeal cancer, Treatment method, Recurrence, Follow-up

## Abstract

**Purpose:**

We assessed the treatment outcome and the benefits of routine follow-up visits in T1 glottic laryngeal squamous cell carcinoma (LSCC).

**Methods:**

Medical records of patients diagnosed with stage T1 glottic LSCC (*N* = 303) in five Finnish university hospitals between 2003 and 2015 were reviewed. Moreover, data from the Finnish Cancer Registry and the Population Register Center were collected.

**Results:**

Of all 38 recurrences, 26 (68%) were detected during a routine follow-up visit, and over half (21 of 38, 55%) presented without new symptoms. Primary treatment method (surgery vs. radiotherapy) was not connected with 5-year disease-specific survival (DSS) or laryngeal preservation rate.

**Conclusion:**

The majority of recurrences were detected on a routine follow-up visit, and local recurrences often presented without new symptoms. Routine post-treatment follow-up of T1 glottic LSCC seems beneficial.

**Trial registration:**

Trial registration number and date of registration HUS/356/2017 11.12.2017.

## Introduction

T1 glottic laryngeal squamous cell carcinoma (LSCC) can be treated either with trans-oral laser surgery or with radiotherapy. The prognosis is excellent regardless of the treatment method [1–8]. In Finland, LSCC is mainly treated in university hospitals. The Finnish Head and Neck Oncology Working Group gives treatment guidelines for head and neck malignancies, and Multidisciplinary Tumor Board in each university hospital gives treatment recommendations for individual patients. Routine follow-up visits for head and neck cancer patients have been scheduled every 3–6 months during the 1st and 2nd year after treatment, and every 6–12 months thereafter, up to 5 years. However, the follow-up protocol varies between university hospitals, and clinicians may intensify or de-intensify follow-up intervals for individual patients, according to their risk profile.

In T1 glottic LSCC, post-treatment surveillance is primarily aimed at early detection of local recurrence. In recurrent head and neck cancer, the presenting symptoms, the pattern of recurrence (locoregional vs. distant), and options available for further treatment (curative vs. palliative) depend on the primary tumor subsite. The previous studies on post-treatment follow-up typically address all head and neck subsites as a single group [9, 10]. Tumor stage and site have a strong impact on likelihood of recurrence. Thus, similar follow-up protocols may not be applicable for all head and neck subsites. Recurrences of T1 glottic LSCC are typically local, and treatment of recurrent disease is often successful.

The aims of our study were to assess nationwide treatment outcome of T1 glottic cancer, with a special attention on the role of routine follow-up visits in detection of cancer recurrence.

## Materials and methods

We included all patients diagnosed with stage T1 glottic LSCC in the five Finnish university hospitals between years 2003 and 2015. Data were collected from the National Cancer Registry, and from medical records in each university hospital. The dates of death were collected from the Population Register Center. The study group comprised 303 patients (mean age 67 years, range 29–93), of whom 263 (87%) were male. All study patients were treated with curative intent, either with surgery or with radiotherapy. Transoral endoscopic surgery was the preferred method. Only one patient underwent open surgery. Radiotherapy was conventional external beam radiation therapy or intensity-modulated radiation therapy. The typical total radiation dose was 66 Gy in 2-Gy fractions. The treatment modality of the patients is decided in Multidisciplinary Tumor Board meeting according to the national treatment guidelines by The Finnish Head and Neck Oncology Working Group. The oncological outcome of the surgery and radiotherapy is considered as equal. Hence, function and individual patient-related factors are regarded in the treatment decisions.

Of all 303 patients, 263 (87%) had a minimum follow-up of 3 years, and 205 (68%) of 5 years, or until death. Routine follow-up visits were programmed every 3–6 months during the 1st and 2nd year after treatment, and every 6–12 months until 5 years. Laryngoscopy using a mirror, a fiber-optic endoscope, or a video-endoscope was the method used for clinical examination during follow-up.

Local recurrence was defined as presence of squamous cell carcinoma in biopsy specimen obtained more than 6 months after treatment of primary disease. Positive biopsy specimens obtained less than 6 months after treatment indicated residual, rather than recurrent, disease. Overall survival (OS) was defined as the duration from diagnosis to death from any cause. Recurrence-free survival (RFS) was defined as the duration from diagnosis to the first documented recurrence. Disease-specific survival (DSS) was defined as the duration from diagnosis to the death caused by LSCC.

The Ethics Committee of Surgery in the Hospital District of Helsinki and Uusimaa approved the study. Institutional permissions were granted. According to the Finnish law, informed consent is not required from patients in a retrospective study design.

IBM SPSS Statistics 24.0 was used for statistical analyses. Chi-square and Fisher’s tests were used to compare categorical variables, and *t *test and Mann–Whitney *U* test to compare continuous variables. Kaplan–Meier method and log-rank test served to compare association between categorical variables and survival. Statistically significant *p* value was set at 0.05. STROBE reporting guideline followed in this study.

## Results

### Clinical characteristics

We included 303 patients with T1 glottic LSCC. As expected, the number of patients diagnosed per university hospital (range 24–130) varied according to their population coverage. Of 303 patients, 274 (90%) had stage T1a, and 29 (10%) T1b disease. Table 1 presents clinical characteristics of all study patients.

**Table 1:** Clinical characteristics of all patients with T1 glottic cancer

Characteristic	*n*	(%)
Male sex	263	(87)
Smoking history		
Yes	256	(85)
No	33	(11)
NA	14	(5)
Earlier dysplasia^a^		
Yes	32	(11)
No	240	(79)
NA	31	(10)
T stage		
T1a	274	(90)
T1b	29	(10)
N stage		
0	302	(100)
1	1	(0)
Primary treatment		
Surgery	163	(54)
Radiotherapy	140	(46)
Recurrence		
Local	35	(12)
Regional or distant	3	(1)
No	265	(87)
Died of LSCC		
Yes	8	(3)
No	295	(97)
Second primary tumor, *n* (%)		
Yes	20	(7)
No	283	(93)

*LSCC* laryngeal squamous cell carcinoma, *NA* not available

^a^Presence of dysplasia confirmed in laryngeal biopsy before diagnosis of T1 glottic cancer

### Primary treatment

In total, 163 (54%) patients were treated with surgery and 140 (46%) with radiotherapy. The proportion of patients treated with surgery (range 13–64%) varied significantly between the five university hospitals (*p* < 0.001).

Patients with T1b LSCC were more likely to undergo radiotherapy than T1a patients (83% vs. 42%, *p* < 0.001, respectively). In T1a tumors, involvement of the anterior third of vocal fold did not correlate with the primary treatment method.

**Fig. 1:**
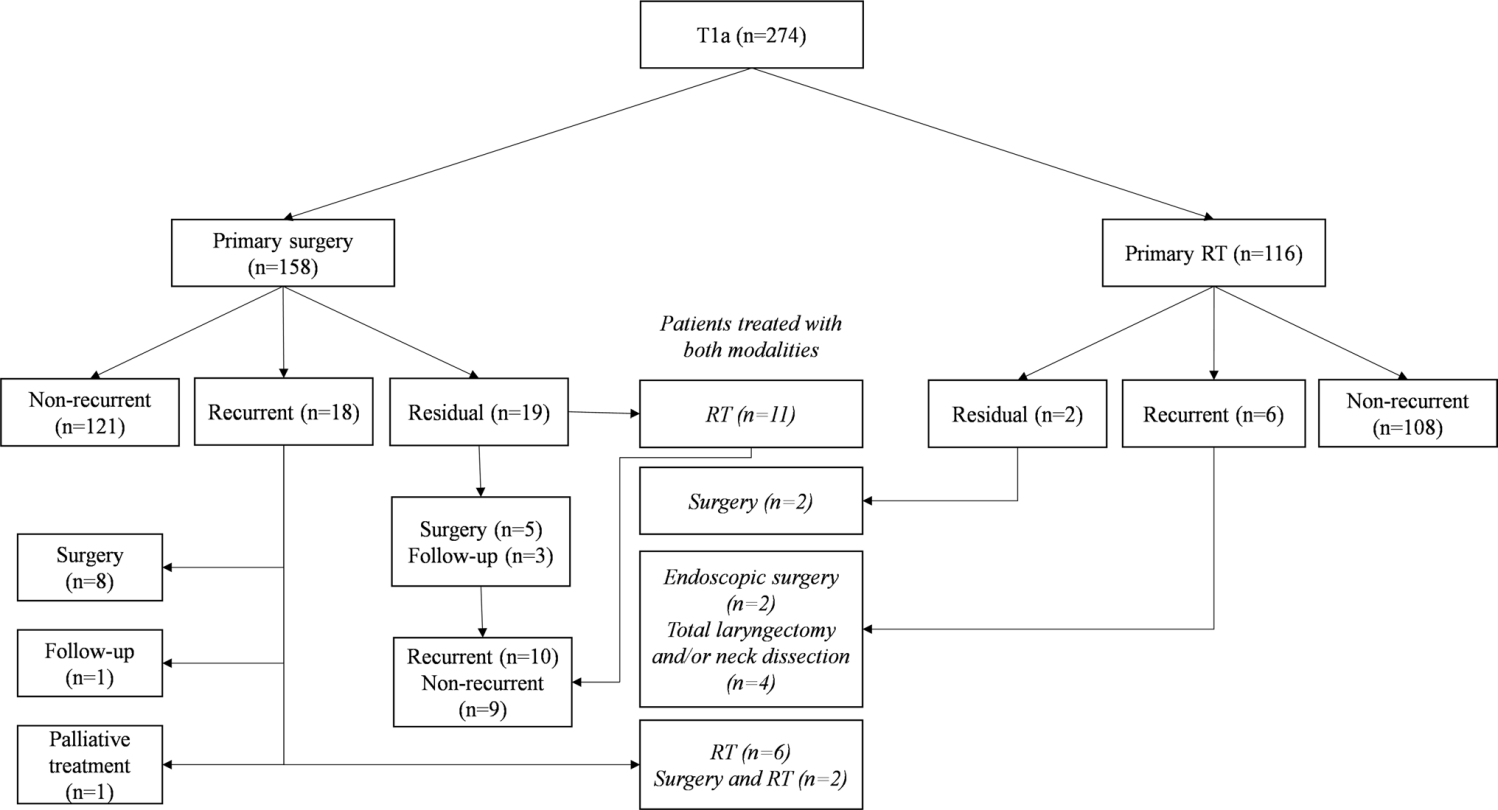
Treatment of 274 T1a LSCC patients. The patients who eventually underwent both surgery and radiotherapy are shown in the middle (in italics). *RT* radiotherapy

Of 163 patients treated primarily with surgery, 11 (7%) had residual tumor which was treated with radiotherapy. Conversely, 2 of 140 (1%) patients primarily treated with radiotherapy had residual tumor which was treated with endoscopic surgery or total laryngectomy and neck dissection. The treatment of T1a patients is presented in the Fig. 1.

### Recurrences

In total, 38 of 303 patients (13%) with T1 LSCC developed a recurrence during follow-up (Table 2). Of 38 recurrences, 35 (92%) were local and 3 (8%) regional or distant. The median time between primary LSCC diagnosis and local recurrence was 1.7 years (range 0.6–8.9 years). Of 35 local recurrences, 7 (20%) were diagnosed more than 5 years after primary LSCC diagnosis.

**Table 2:** Patients with recurrence (*n* = 38)

Characteristic	All patients with recurrence (*n* = 38)	Time interval from diagnosis to recurrence		
		< 2 years (*n* = 23)	≥ 2 years (*n* = 15)	*p*
Male sex, *n* (%)	29 (76)	17 (74)	12 (80)	1.000
Primary treatment, *n* (%)				
Surgery	29 (76)	18 (78)	11 (73)	1.000
Radiotherapy	9 (24)	5 (22)	4 (27)	
Recurrence detected on a routine follow-up visit, *n* (%)				
Yes	26 (68)	19 (83)	7 (47)	0.033*
No	12 (32)	4 (17)	8 (53)	

*Statistically significant

Local recurrence rates in T1a (31 of 274, 11%) and T1b (4 of 29, 14%) were similar (*p* = 0.758). University hospitals had similar local recurrence rates (*p* = 0.340).

Five-year RFS was 91% in all patients, 91% in T1a patients and 86% in T1b patients. The corresponding numbers for 3 year local control were 92, 92 and 90%. RFSs were not associated with T stage (*p* = 0.831) in log-rank test. Presence of earlier dysplasia, age at diagnosis or smoking history was not significantly associated with recurrences, laryngeal preservation or second primary tumor.

### Local recurrences and primary treatment of T1a glottic cancer

Local recurrence of T1a after primary surgery occurred in 25 of 158 (16%), and after primary radiotherapy in 6 of 116 (5%). Thus, local recurrence of T1a was significantly more likely after surgery than radiotherapy (*p* = 0.006). However, 5 of 25 (20%) local recurrences of T1a after surgery occurred over 5 years after primary treatment. Five-year local control in T1a was higher after radiotherapy than surgery (97% vs. 87%, *p* = 0.020).

### Resection margins

Positive resection margins were reported in 15 of 158 (9%) T1a patients treated with surgery, and 7 of them underwent re-resection. Local recurrence was detected in 2 of the 7 (29%) re-operated patients during follow-up, and in 3 of 8 (38%) patients without re-operation. Resection margins were defined as negative if they were not infiltrated by tumor cells, or if negative resection surface biopsy specimens were obtained after endoscopic removal of tumor. Negative resection surface margins were reported in 71 of 158 (45%) T1a patients treated with surgery, and 7 of 71 (10%) developed a local recurrence. Resection surface margin status was not reported for 72 (46%) patients. T1a patients with positive resection margins had higher local recurrence rate than patients with negative margins (33% vs. 10%, *p* = 0.031). Margin status was not associated with DSS in T1a patients (*p* = 0.064).

### Treatment of recurrent T1 glottic cancer

Of all 38 patients with recurrent disease, 14 (37%) underwent radiotherapy, 11 (29%) endoscopic surgery, and 9 (24%) total laryngectomy and/or neck dissection. Total laryngectomy rate was similar in patients primarily treated with surgery (5 of 163, 3%), compared to those treated with radiotherapy (8 of 140, 6%, *p* = 0.395). Two of 38 (5%) patients had palliative care and died in laryngeal cancer after their first recurrence, and two (5%) patients died of other causes.

### Follow-up

**Fig. 2:**
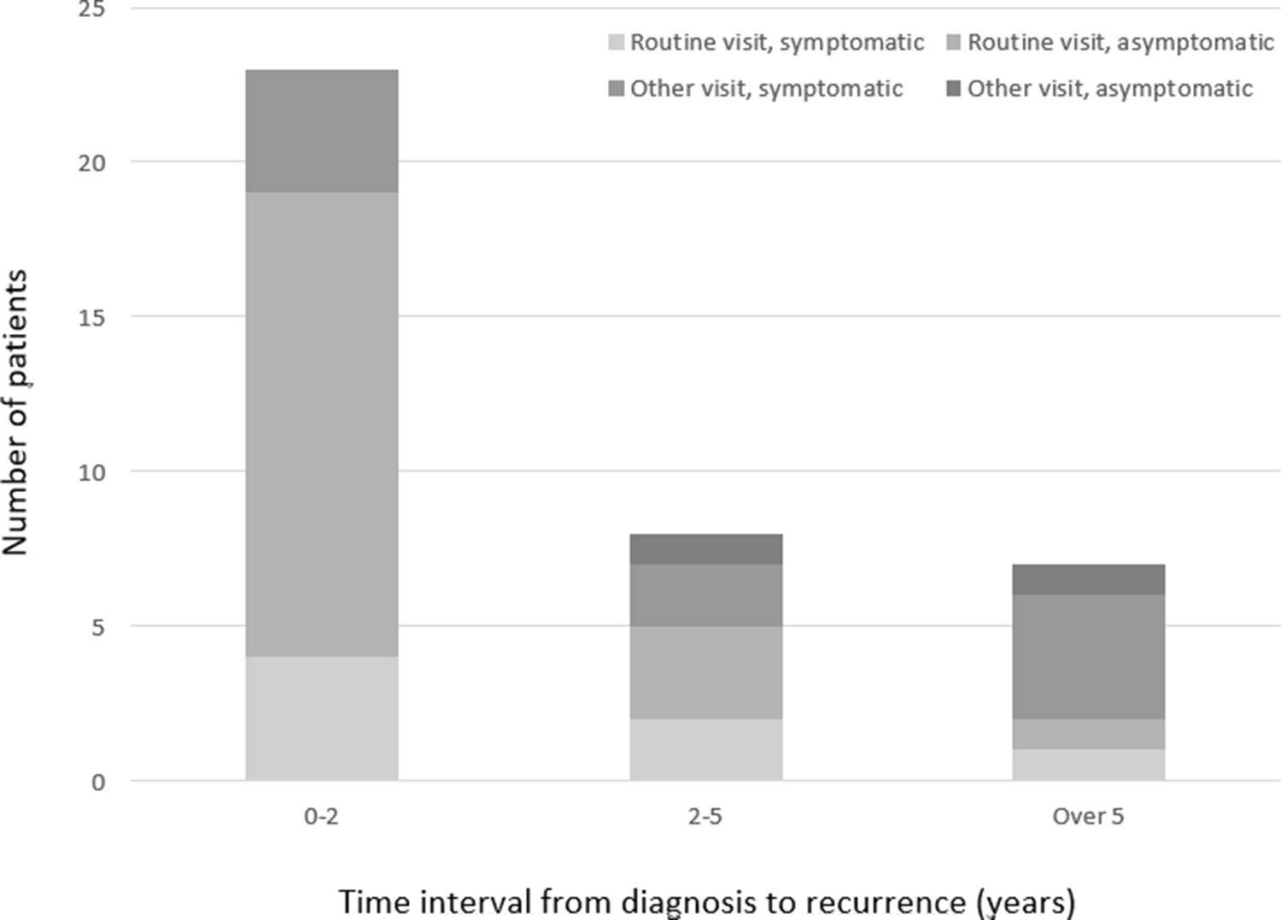
Of 38 T1 LSCC recurrences, 26 (68%) were detected on a routine follow-up visit

The majority of recurrences (26 of 38, 68%) were detected by clinical examination on a routine follow-up visit (Fig. 2). Nine patients (24%) requested a visit because of new symptoms. Two (5%) recurrences were diagnosed during clinical examination of another disease. In one patient (3%), the recurrence was detected by PET imaging, which was part of the follow-up protocol in one university hospital.

Over half (21 of 38, 55%) of recurrences were detected in patients presenting without new symptoms. The most common symptom was hoarseness in 13 patients, followed by pain in 3 and dysphagia in 3. The patients primarily treated with radiotherapy reported pain as a symptom of local recurrence more often compared to patients treated with surgery but the difference was not statistically significant (2 of 9 (22%) vs. 1 of 26 (4%)). Sex or age was connected neither with symptoms of local recurrence, nor with detection of local recurrence on a routine visit versus on other visit. Twenty-one of 31 (68%) T1a patient’s and 3 of 4 (75%) T1b patients’ local recurrences were detected on a routine follow-up visit (*p* = 1.000). Seventeen (55%) of 31 T1a patients and 3 (75%) of 4 T1b patients with local recurrence were symptomatic (*p* = 0.619). The time interval from diagnosis to local recurrence in patients presenting with a new symptom was significantly longer, compared to patients without new symptoms (median 2.7 years (range 0.9–8.4 years) vs. median 1.6 years (range 0.6–8.9 years), Mann–Whitney *p* = 0.019).

The larynx preservation rate was not connected with symptoms of local recurrence, or with detection of local recurrence on a routine follow-up visit. Larynx preservation rate was 60% in the patients with symptomatic local recurrence and 70% in the asymptomatic patients (*p* = 0.721). Accordingly, 9 of 20 (45%) symptomatic local recurrences and 3 of 15 (20%) asymptomatic local recurrences were treated with trans-oral endoscopic surgery.

### Survival of patients with stage T1 glottic cancer and second primary tumors

Of 303 patients, 8 (3%) died of LSCC. Five-year overall survival (OS) of all study patients was 82%, 82% in patients with stage T1a disease and 76% in T1b. The corresponding numbers for 5 year DSS were 98, 97 and 100%. In log-rank test, OS and DSS did not correlate with T stage, university hospital or primary treatment method.

Second primary tumor (either in the head and neck or in the lungs) was diagnosed in 20 of 303 (7%) patients during follow-up. Second primary subsites were lungs (12/20, 60%), hypopharynx (4/20, 20%) and other head and neck subsite or multiple subsites (4/20, 20%). Eight (8/20, 40%) of the second primary tumors were diagnosed concurrently with LSCC, or during LSCC treatment, and 4 (4/20, 20%) more than 5 years after LSCC diagnosis. Of 303 patients, 9 (3%) died of second primary tumor. The primary treatment method was not related to second primary tumors (*p* = 0.248).

## Discussion

We assessed the treatment outcome and the benefits of routine follow-up in T1 glottic LSCC. In our study, most of the recurrences were local, and 26 of 38 (68%) were diagnosed during a routine follow-up visit. Only 45% of patients had new symptoms when the recurrence was diagnosed. Finland has a publicly funded health care system. The role of routine visits is currently discussed to improve cost-effectiveness of follow-up protocols. According to our study, routine post-treatment follow-up of T1 glottic LSCC seems beneficial. Furthermore, clinical follow-up of LSCC with modern flexible endoscopes is easy and feasible, without causing exposure to radiation.

Our results are similar compared to the study by Ritoe et al. where they included LSCC patients with T stage I to IV. In their study, 122 of 156 (78%) LSCC recurrences and second primary tumors were found within 3 year follow-up, and 94 of 156 (60%) recurrences and second primary tumors were detected on a routine follow-up visit [11]. In another study, 81% (103 of 127) of patients with stage I to IV glottic LSCC who developed a recurrence were symptomatic [12]. In our study of T1 patients, the number of symptomatic patients was substantially lower.

After treatment of T1 glottic LSCC, patients may adapt to often permanent changes in voice quality. Some patients may have hoarse voice due to a large post-surgical glottal gap, or mucosal edema after radiation therapy. The first symptom of local recurrence in these patients may be airway obstruction and stridor. Thus, detecting a recurrence of early glottic LSCC by symptom-directed surveillance only is more complicated compared to some other head and neck cancer subsites, in which the majority of recurrences are detected when new symptoms appear [13]. Early detection of local recurrence allows curative treatment with acceptable oncological and functional outcome. Compared to patients with late recurrences, patients with early (< 2 years) recurrence of T1 LSCC were less likely to present with a new symptom, and less likely to be diagnosed on a patient-requested visit. It may take long for the post-treatment voice problems to resolve, and newly treated patients especially may not recognize subtle changes in voice as a symptom of recurrence. Furthermore, patients may not request for an extra visit because follow-up during the first 2 years is typically intense.

The 5-year RFS and DSS in this study were comparable to the previous review studies [5, 8, 14, 15]. A Finnish nationwide study showed that 5-year RFS in T1a glottic cancer was 88%, and DSS 100%. In T1b glottic cancer, RFS was 82% and DSS 95% [16]. Our previous prospective randomized study of 56 patients showed that T1a glottic LSCC recurred in 11%, already during 2-year follow-up [17].

Five out of 303 (1.7%) patients developed glottic LSCC over 5 years after primary diagnosis. We considered these as recurrences rather than new primary tumors. The definitions of treatment failure, recurrence, and new primary tumor are somewhat controversial. According to the previous literature, late cancer events in LSCC are rare [18]. Prolonged post-treatment follow-up may not be justified in all patients with T1 glottic LSCC. If new symptoms should occur, the threshold for re-examination should be low.

Primary surgery was associated with inferior RFS, compared to primary radiotherapy. Radiotherapy has earlier been the gold standard for T1 LSCC treatment but in surgery a major change occurred after an article by W. Steiner in 1993 [19]. Endoscopic laser surgery of early LSCC was introduced soon also in Finland, and there may have been surgeon-related learning curve effect in the beginning. In addition, possible errors in T-staging could have affected the results. However, we found no association between the primary treatment method and the other outcome measures, such as DSS and OS. Moreover, larynx preservation rate was similar in both treatment methods. Selection of the primary treatment varied significantly between the five university hospitals. Radiotherapy was preferred in university hospitals where the number of patients was smaller. Possibly, in these smaller units, the availability of radiotherapy was better compared to surgery.

Our study represents approximately 60% of T1 LSCC cases diagnosed in Finland between years 2003 and 2015, because patients treated in non-academic centers were not included [16, 20]. Complete follow-up data were unavailable for some patients, since they were treated in the smaller university hospitals, responsible for large geographical areas with long distances. Soon after treatment, these patients were referred to non-academic hospitals for follow-up. Due to the retrospective study design, detailed information was not always available for smoking, previous dysplasia, margin status, and presence of new symptoms in patients presenting with local recurrence.

The National Cancer Database study from the USA assessed positive margin rates in trans-oral laser microsurgery of LSCC. Cases treated at non-academic centers and those with lower caseloads had a higher likelihood of positive margins [21]. Silverman et al. showed that postoperative radiotherapy of T1-T2 LSCC was significantly more common in low-volume centers [22]. In our study, positive margin status in T1a was associated with a higher risk of local recurrence. Furthermore, 11 of 163 (7%) patients primarily treated with surgery had residual tumor, requiring further treatment with radiotherapy. These findings highlight the need for thorough pre-treatment work-up and surgical skill, since bimodal treatment of T1 LSCC should be avoided. In our retrospective study, margin status was unavailable for a large number of patients (46%). The prognostic value of positive margin status, according to the literature, is controversial [23, 24]. A uniform guideline for determining negative resection margins would be useful, and margin status should be considered in upcoming recommendations for clinical follow-up.

Pre-treatment diagnostic work-up is the key element in choosing the optimal treatment modality. RFS was inferior in patients primarily treated with surgery, compared to patients treated with radiation therapy. However, DSS, OS and laryngeal preservation rate were similar. Treatment of T1 glottic LSCC may cause permanent voice changes, and symptoms of local recurrence can be misinterpreted as post-treatment sequelae. Routine post-treatment follow-up of T1 glottic LSCC is beneficial.

## Data Availability

The datasets used and/or analyzed during the current study are available from the corresponding author on reasonable request.
